# The Tissue Factor Pathway in Cancer: Overview and Role of Heparan Sulfate Proteoglycans

**DOI:** 10.3390/cancers15051524

**Published:** 2023-02-28

**Authors:** Nourhan Hassan, Janes Efing, Ludwig Kiesel, Gerd Bendas, Martin Götte

**Affiliations:** 1 Department of Gynecology and Obstetrics, Münster University Hospital, Domagkstrasse 11, 48149 Münster, Germany; 2 Biotechnology/Biomolecular Chemistry Program, Faculty of Science, Cairo University, Giza 12613, Egypt; 3 Pharmaceutical Department, University Bonn, An der Immenburg 4, 53225 Bonn, Germany

**Keywords:** tissue factor, platelets, proteoglycans, cancer, syndecans

## Abstract

**Simple Summary:**

Tissue factor is a protein that is important for the regulation of blood coagulation. New research has highlighted the important roles of tissue factor in cancer. This review summarizes recent work that shows how a special class of glycoproteins called heparan sulfate proteoglycans regulate tissue factor function in a cancer context. These findings provide new ideas for future anti-cancer therapies.

**Abstract:**

Historically, the only focus on tissue factor (TF) in clinical pathophysiology has been on its function as the initiation of the extrinsic coagulation cascade. This obsolete vessel-wall TF dogma is now being challenged by the findings that TF circulates throughout the body as a soluble form, a cell-associated protein, and a binding microparticle. Furthermore, it has been observed that TF is expressed by various cell types, including T-lymphocytes and platelets, and that certain pathological situations, such as chronic and acute inflammatory states, and cancer, may increase its expression and activity. Transmembrane G protein-coupled protease-activated receptors can be proteolytically cleaved by the TF:FVIIa complex that develops when TF binds to Factor VII (PARs). The TF:FVIIa complex can activate integrins, receptor tyrosine kinases (RTKs), and PARs in addition to PARs. Cancer cells use these signaling pathways to promote cell division, angiogenesis, metastasis, and the maintenance of cancer stem-like cells. Proteoglycans play a crucial role in the biochemical and mechanical properties of the cellular extracellular matrix, where they control cellular behavior via interacting with transmembrane receptors. For TFPI.fXa complexes, heparan sulfate proteoglycans (HSPGs) may serve as the primary receptor for uptake and degradation. The regulation of TF expression, TF signaling mechanisms, their pathogenic effects, and their therapeutic targeting in cancer are all covered in detail here.

## 1. Introduction

Blood coagulation generally serves as a host defense mechanism against bleeding. The corresponding coagulation cascade is triggered upon vessel wall perforation or activation of the endothelium by chemicals, cytokines, or inflammatory processes [1,2,3,4,5,6]. A vascular lesion is initially occluded by a platelet plug whose formation is coordinated through the coagulation system response [6]. The blood coagulation system presents as a complex, highly regulated process with the participation of more than a dozen plasma proteins, at least one tissue protein, phospholipid membrane surfaces, Ca^2+^ ions, and platelets [7]. In normal physiology, the hemostatic system maintains the circulating blood in a fluid state; several anticoagulant mechanisms ensure careful control of coagulation by prevailing the procoagulant forces explained in the following that allow a rapid response to vessel wall rupture [6,8].

## 2. Tissue Factor—Structure and Expression Profile

Tissue factor (TF), or coagulation factor III, is a cell surface glycoprotein of 47 kDa comprising a 23 residue transmembrane domain flanked by a short cytoplasmic tail and a relatively large N-terminal extracellular domain (ECD) [9,10,11,12]. Its function as the primary initiator of physiological hemostasis is closely related to its particular expression pattern within the human body. TF is not expressed equally in all tissues; the highest expression was found in the brain, lung, epithelial cells of the skin, heart, and testis [13,14,15,16,17,18]. Besides the expression levels in these specific organs, TF levels can be found on subendothelial surfaces, in particular, adventitial fibroblasts of large blood vessels, and pericytes surrounding smaller vessels [17]. Cells directly exposed to flowing blood, such as endothelial cells, normally do not express any TF [10]. Following this circumstance, the ‘hemostatic envelope’ theory was postulated, which emphasizes the restriction of TF expression to certain cell types to allow initiation of the coagulation cascade only when vascular integrity is breached [13]. Besides the subendothelial TF populations, there are also circulating TF molecules known as blood-borne TF [19]. They circulate within the bloodstream in at least three different pools: (1) associated with white blood cells and platelets [20,21], (2) located on cell-derived microparticles (a subclass of extracellular vesicles) [22], or (3) as a soluble protein [23]. The latter—also known as alternative spliced TF (asTF)—lacks the transmembrane domain as well as the cytoplasmic tail, and therefore displays no membrane association [23]. Healthy individuals’ plasma mean level of blood-borne TF ranges between 149 and 172 pg/mL [24,25].

## 3. Coagulation Factors and Blood Clotting

**Figure 1:**
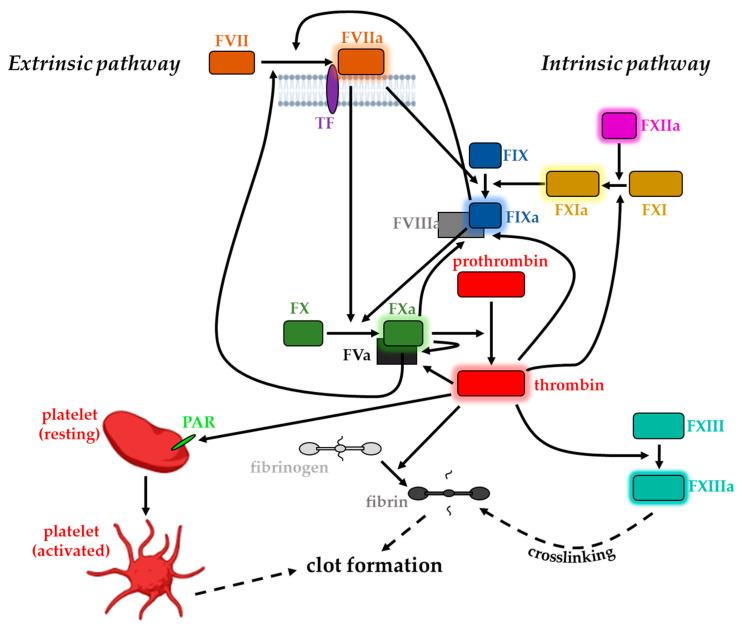
Schematic overview of the coagulation cascade: Extrinsic pathway is initiated by the binding of FVIIa to TF, intrinsic pathway involves activation of FXIa; both pathways finally merge, resulting in thrombin generation which fulfills several functions required for efficient clot formation. Adapted from BioRender.com (accessed on 16 May 2022).

The blood clotting cascade can be subdivided into two major pathways: the extrinsic and the intrinsic. The nomenclature of the former originates from the fact that its initiation involves direct contact of blood plasma with ‘something extrinsic’ with respect to the vasculature [26]. The extrinsic pathway initially involves a high-affinity association of subendothelial TF with plasma protein FVII and its activated form (FVIIa), respectively [27]. FVIIa displays a serine protease whose activity is heavily enhanced (2 × 10^7^-fold higher enzymatic activity rate) [28] when associated with TF due to active site region rearrangements [29,30,31] (Figure 1). FVIIa is synthesized in the liver and circulates in human blood at concentrations of approximately 10 nM [32], to a great extent in its inactivated form. Exposure of TF following vessel injury and blood extravasation leads to complexation with plasma FVII. Subsequent activation of the protease [33] affects two major downstream substrates in the coagulation cascade via limited proteolysis [26]. In the first place, the protein complex promotes the conversion of minute quantities of FX, another plasma serine protease, to its activated form (FXa). Secondly, the circulating FIX serine protease zymogen is also converted to an active conformation (FIXa) [34,35]. However, besides the physical proximity, the full activity of the TF/FVIIa tandem to activate its downstream targets requires the presence of a membrane surface as well as Ca^2+^ ions [10]. FXa and FIXa must assemble on a suitable membrane surface together with their cofactors: FVa and FVIIIa, respectively [26]. The so-generated small amounts of FXa participate in the prothrombinase complex with its cofactor FVa and serve as the primary activator of prothrombin by catalyzing the locally restricted proteolytic cleavage of prothrombin to thrombin [6,11,36,37,38,39]. Feedback amplification at several levels is crucial for the efficiency of the cascade: TF-bound FVII gets activated to FVIIa, FIXa, and FXa. FXa itself, when membrane-bound, in turn, can also activate its own cofactor FV as well as FVIII (Figure 1) [40]. Additionally, thrombin-dependent activation of FXI, FVIII, and FV occurs, ultimately resulting in a rapid burst in thrombin generation [6,41,42,43,44,45].

All components participating in the alternative intrinsic pathway can be found solubilized in blood plasma. Thus, its initiation requires no extravascular molecule, making it TF-independent; consequently, activation of the intrinsic blood coagulation pathway does not necessarily involve any trauma [46]. Initially, FXIIa converts FXI to its activated form (FXIa). FXIa, in the presence of Ca^2+^, increases FIXa level, which is membrane-associated due to the binding of its cofactor FVIIIa. Activated FIX in turn is capable of proteolytically activating FX, finally leading to the same downstream result of thrombin generation [28,46,47]. However, despite the similarities in downstream signaling to its extrinsic counterpart, the intrinsic pathway is not inevitably essential for normal blood coagulation [46]. FXa integrates the two pathways and therefore shows up as a critical point of the coagulation process: the serine protease receives extrinsic and intrinsic upstream signals and orchestrates downstream responses [27]. Several protease components are partially involved in both pathways, emphasizing them to be highly interconnected. Generally, the plasma concentrations of the particular coagulation proteins relate to their specific role in the corresponding pathway; while the early components of the pathway circulate in a less great abundance, factors more downstream can be found in higher quantities [48,49]. 

Both the extrinsic and the intrinsic pathways finally result in the locally restricted generation of thrombin from its zymogen prothrombin. Thrombin exhibits multifaceted functions and is regarded as the key enzyme in the coagulation cascade due to the affection of several downstream targets [50]. It displays a Na^+^-activated allosteric serine protease that is assigned to the chymotrypsin family [51] with its zymogen predominantly synthesized in the liver, the major site of clotting factor synthesis [50,52,53,54]. Thrombin is mainly responsible for the initiation and propagation of clot formation: its procoagulant effect relies on its capability to cleave plasma fibrinogen into an insoluble fibrin clot that directs and anchors activated platelets to the site of the lesion [46,55,56]. The establishment of the clot composed of fibrin molecules and activated platelets is further reinforced by the activation of FXIII, also by thrombin. FXIIIa activity results in covalent fibrin crosslinking, which in turn stabilizes the assembled clot [11,48,57]. 

In addition to its role as a blood coagulation trigger, TF associated with FVIIa can activate members of the protease-activated receptors (PAR) family [58]. This activity seemingly depends on the catalytical activity of FVIIa and has been considered with respect to several pathophysiological scenarios [58]. The unique, cleavage-triggered activation of PARs is normally associated with thrombin receptor activity. In these terms, thrombin can activate platelet via PARs on platelet surfaces. Thus, thrombin initiates various intracellular signaling events finally resulting in the transformation of the normally mobile, non-adhesive platelets into central participants of hemostatic clot growth primarily via morphological changes [55,59,60]. Apart from their participation in clotting, these transformed platelets also provide binding sites for the different serine protease–cofactor complexes that assemble due to the propagation of the coagulation cascade [43].

## 4. Regulation of Tissue Factor and Blood Coagulation

Interestingly, besides its procoagulant role in blood clotting, thrombin once generated in blood also shows some opposing anticoagulant activities. Upon binding to endothelial membrane receptor thrombomodulin, the proteases’ ability to cleave fibrinogen and PAR1 is suppressed, while simultaneously its specificity toward zymogen protein C is massively enhanced (>1000-fold) [61,62]. The activated protein C proteolytically cleaves and thereby inactivates FVa and FVIIIa, the essential cofactors of the coagulation cascade proteins FXa and FIXa, respectively, required for efficient thrombin generation [63]. By this mechanism, downregulation of both the amplification and progression of the coagulation cascade occurs, emphasizing the versatile role of thrombin in coagulation [64]. In normal hemostasis, the thrombin–thrombomodulin tandem with subsequent protein C activation constitutes a natural anticoagulant pathway to prevent severe intravascular emergence of a fibrin clot upon thrombin hyperactivity [64,65]. 

Generally, regulation of the coagulation cascade occurs at each level, either by enzyme inhibition or modulation of the cofactor activity. Endogenous inhibition of the TF pathway has been known since the 1950s [66] and the accountable molecule that inhibits the initiation phase of extrinsic blood coagulation was identified approximately 30 years later [67,68,69,70,71]. 

Tissue factor pathway inhibitor (TFPI) appears as a high-affinity inhibitor of relatively low abundance in blood plasma [67,72]. It directly suppresses FXa activity and further also TF/FVIIa activity in an FXa-dependent manner [72,73,74,75,76,77]. There are two major isoforms of TFPI based on alternative splicing: TFPIα and TFPIβ [71,78,79]. They differ in their tissue expression pattern [80,81,82] and their cell surface association mechanisms [79,83,84,85], as well as regarding their ability to affect TF/FVIIa and prothrombinase activity, respectively [76,86]. While TFPIα is mainly associated with plasma membranes of the corresponding target cells via heparan sulfate proteoglycans [87,88] or with low-density lipoprotein (LDL) when solubilized in blood plasma [6], TFPIβ comprises a GPI anchor sequence and thus is directly bound to the surface of endothelial cells [78,89]. In humans, the existence of a third minor isoform, namely, TFPIδ, has been discovered [90]; however, its role is much less explored. Besides protein C and TFPI, a third major inhibitor warrants the tight regulation of blood clotting: antithrombin (antithrombin III). Antithrombin acts as a serine protease inhibitor suppressing the activity of FXa and other protease components of the cascade [91]. It is postulated to be the main inhibitor of thrombin and its generation [42] 4. However, to efficiently fulfill its inhibitory function, antithrombin needs to act in consultation with heparin and heparin-like molecules on the surface of endothelial cells [92,93,94,95,96,97]. Heparin also accelerates the action of TFPI [98]. In vitro, TFPI and heparin have been shown to have synergistic inhibitory activity on TF-induced coagulation [99,100]. 

These different inhibitory mechanisms partially act in synergy with the consequence of thrombin generation being a threshold-limited process. They also feature some interconnections [36,101,102,103]. For instance, protein S acts as a cofactor for both protein C and TFPI [104]. A final and not less important anticoagulant mechanism comprises the fibrinolytic system whose enzymes finally dissolve the clot after it accomplishes its function [46].

The importance of the numerous inhibitory components for the health of individuals is undisputed. First, this is substantiated by the fact that there is no common human TFPI deficiency, suggesting it results in embryonic lethality [105]. Furthermore, deficiencies of other essential inhibitory components of the coagulation system such as protein C or antithrombin are known as one main cause of thrombosis due to excessive thrombin generation [106]. On the other hand, thrombosis might also occur as a consequence of elevated concentrations of particular clotting factors common for certain liver diseases [107,108]. 

On the other hand, in various cell types and tissues, many miRNAs have been found to directly modulate TF expression and thus its functions. This can have an impact on both physiological and pathological processes [109]. MiR-19b and miR-20a, for example, suppress TF expression in vitro and are observed to be reduced in monocytes obtained from patients with systemic lupus erythematosus and antiphospholipid syndrome. This suggests that miRNA control of TF may play a role in these disorders [110]. According to one study, miR-126 has antithrombotic characteristics by affecting the hemostatic equilibrium of the vasculature in diabetes mellitus by modulating TF expression. MiR-126 can be a predictive biomarker for diabetes mellitus progression and complications [111]. MiR-145 levels were restored in thrombotic animals via in vivo miR-145 mimic treatment, which resulted in lower TF levels and activity, as well as lower thrombogenesis. MiR-145 levels were likewise lowered in individuals with venous thromboembolism (VTE) and were linked to higher TF levels. Notably, when endothelial cells (ECs) were transfected with a miR-223 mimic or inhibitor, TF expression was changed accordingly at both the mRNA and protein levels. On damaged atherosclerotic plaques, TF initiates thrombosis, which is important during the diagnosis of acute coronary syndromes (ACS). In atherosclerosis, overexpression of miR-223 decreased TF procoagulant activity.

The potential of the TF-FVIIa complex to initiate coagulation is defined as TF procoagulant activity (PCA). Detergents, Ca^2+^ ionophores, and oxidants are among the agents that can greatly increase TF PCA [112,113,114]. Previous studies reported that the elevation in TF PCA associated with oxLDL treatment also stimulates the transcription of TF mRNA, whereas treatment with hydrogen peroxide (H_2_O_2_) only elevates TF PCA [115]. Since so much expressed TF is accessible on the cell surface, many regulatory pathways are required to avoid spontaneous broad coagulation [116].

## 5. TF/FVIIa Intracellular Signaling

**Figure 2:**
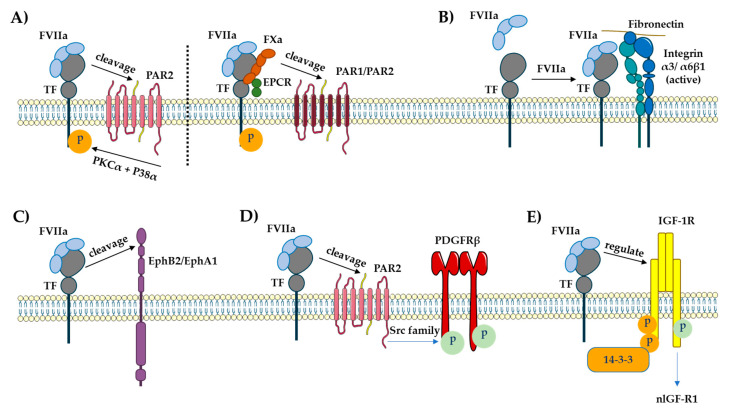
Cell surface-bound full-length TF interacts with a number of different proteins in order to initiate signaling and achieve specificity. (**A**) PAR1 is cleaved by the TF/FVIIa/FXa complex whereas PAR2 is cleaved by both TF/FVIIa and TF/FVIIa/FXa. TF cytoplasmic domain is Ser253 phosphorylated by PKCα and Ser258 phosphorylated by P38α. (**B**) Association of TF with β1 integrins is regulated by TF extracellular ligand binding and independent of PAR2 signaling or proteolytic activity of VIIa. (**C**) EphB2 and EphA2 are proteolytic targets of TF/FVIIa. (**D**) TF/FVIIa transactivates the PDGFRβ in a PAR2 and Src family. (**E**) TF/FVIIa transactivates IGF-1R and mediates the signaling cascades generated by IGF-1 stimulation. Parts of the figure were drawn using elements from Servier Medical Art. Servier Medical Art by Servier is licensed under a Creative Commons Attribution 3.0 Unported License (https://creativecommons.org/licenses/by/3.0/) (accessed on 16 May 2022).

TF serves as a signaling receptor in addition to its action in coagulation [58]. Rottingen et al. reported this evidence in 1995 when they found that adding FVIIa to a variety of TF-expressing cell types triggered Ca^2+^ oscillation [117]. Both TF/FVIIa complex and TF/FVIIa/FXa complex can mediate TF signaling. By activating integrins and transactivating or proteolytically cleaving certain members of the receptor tyrosine kinase (RTK) family, signaling can be dependent or independent of PAR2 and PAR1 (Figure 2) [118]. 

Mitogen-activated protein kinases (MAPKs) including p44/42, p38, and C-Jun N-terminal kinase (JNK), which are responsible for cell cycle control, and PI3K/AKT, which are mainly essential for cell survival, are among the signaling components identified to be activated by TF/FVIIa [119,120]. Src-family kinases are involved in the upstream processes of both p44/42 and PI3K/AKT [121,122]. Since cell survival and migration, as well as inflammation and angiogenesis, are key processes in cancer cell progression and tumor development, the impact of TF/FVIIa signaling in oncology is apparent [123,124,125,126,127] and will be discussed in detail in this review. 

### 5.1. PAR Signaling

When the thrombin receptor was cloned in 1991, the PAR family of G protein-coupled receptors (GPCRs) was discovered [128]. In humans, four recognized PARs are extensively expressed in a variety of cell types. While GPCRs are usually activated by endogenous extracellular agonists, PARs are cleaved particularly near the N-terminus by proteases unmasking a tethered ligand, which subsequently folds back to activate the receptor (Figure 2A) [128]. Although PAR1 is the most common thrombin receptor, thrombin can also cleave and activate PAR3 and PAR4. The TF/FVIIa/FXa complex can cleave and activate PAR1 in addition to thrombin. In a process independent of the TF cytoplasmic domain, the cleavage of PAR1 by TF/FVIIa/FXa can result in Ca^2+^ influx and p44/42 MAPK activation [127,129,130]. PAR2 is thrombin-insensitive, unlike the other PARs, but can be induced by direct cleavage by both TF/FVIIa or the TF/FVIIa/FXa complex. Furthermore, the cytoplasmic domain of TF may be required for PAR2 signaling [131,132]. While low concentrations of FVII are required for cleavage of PAR1/PAR2 via TF/FVIIa/FXa complex as for coagulation activation, much greater amounts of TF/FVIIa complex are required to activate PAR2 (Figure 2A) [133,134]. Signaling can also be dependent on the phosphorylation of the TF cytoplasmic domain when PAR2 is activated by the TF/FVIIa complex [135]. The activation of PAR2 by TF/FVIIa results in the release of cytokines and angiogenic factors that are important in inflammatory, gastrointestinal, respiratory, cardiovascular, metabolic, and neurological illnesses, as well as malignancies [136]. Furthermore, insulin resistance and inflammation in adipose tissue have also been linked to TF-PAR2 signaling in hematopoietic and myeloid cells [137]. 

### 5.2. Receptor Tyrosine Kinase (RTK) Signaling

RTKs are extracellular ligand receptors that are distinguished by their intrinsic tyrosine kinase activities. Ligand interaction causes receptor subunit dimerization and tyrosine kinase moiety activation, followed by the transphosphorylation of tyrosine residues in the cytoplasmic domain of the receptor [138]. These tyrosines serve as binding sites for the adapter proteins with SH2- and PTB-domains that induce downstream signaling via the Ras/MAPK or PI3K/Akt pathways [139]. Growth factors and hormones, which are required for cell differentiation, proliferation, and motility, serve potential RTK ligands. RTK signaling is essential for cell development and function, and also plays a role in the pathophysiology of many human disorders [140]. The uncontrollable proliferation of tumor cells is driven by deregulated growth factor signaling, which constitutes a fundamental event in many malignancies [141]. The Eph tyrosine kinase receptors constitute the biggest RTK family in the human genome, comprising 14 members [142]. Thereof, EphB2 and EphA2 have been identified as TF/FVIIa proteolytic targets. The ectodomains of these receptors can be cleaved by TF/FVIIa, affecting Eph-mediated cell division and segregation (Figure 2C) [143]. In addition, TF can act as a molecular ligand directly interacting and thus activating RTKs. EGF receptor (EGFR), PDGF receptor β (PDGFRβ), and insulin-like growth factor 1 receptor (IGF1R) are known to be transactivated by TF/FVIIa [144,145,146]. The triple membrane-spanning model (TMSP) describes how the EGFR is transactivated by stimulating metalloproteinases, which then release heparin-bound EGF to activate the receptor. Intracellular protein kinases may also play a role in transactivation. A unique pattern of phosphorylation at four tyrosines in the PDGFRβ cytodomain has been identified as a result of TF/FVIIa-induced transactivation of the PDGFRβ. PAR-2 is also required for this process (Figure 2D) [145]. On the other hand, the transactivation of the IGF-1R is not dependent on PARs [144]. The magnitude of the tyrosine phosphorylation responses by TF/FVIIa is lower than that of the native ligands of the receptor, indicating the receptors to be only partially activated and thus activation to be selective. 

Although the physiological consequences of EGFR transactivation are less obvious, TF/FVIIa transactivation of PDGFRβ and IGF-1R has a significant influence on the target cells. Due to the activation of Src-family kinases and the transactivation of PDGFRβ, TF/FVIIa stimulates smooth muscle cells, fibroblasts, monocytes, and endothelial cells to migrate towards a 100-fold lower concentration difference of PDGF-BB than cells without TF/FVIIa complex formation [146]. 

### 5.3. IGF-1R Signaling and the Tissue Factor Pathway

The insulin and IGF-1 receptor families have a common ancestor and display a lot of similarities. Their roles have been merged over time, where the insulin receptor mainly regulates glucose metabolism and the IGF-1R regulates cell proliferation and growth [147]. The ligands IGF-1 and IGF-2, as well as insulin at high doses, activate the IGF-1R [148]. Most cells in the body express IGF-1R, and it is also frequently (over)expressed in neoplastic cell lines and human tumors [149]. IGF-1R is a tetramer made up of two α- and two β-subunits that are linked together by disulfide bonds [150]. When a ligand binds to the α-subunit of the receptor, three tyrosine residues Tyr1131, Tyr1135, and Tyr1136 in the activation loop of the kinase domain are auto-phosphorylated. Phosphorylation of other residues in the β-subunit serves as docking sites for several proteins including insulin receptor substrate (IRS), which mediates the signaling cascades generated by IGF-1 stimulation (Figure 2E) [150,151]. IGF-1R signaling has also recently gained attention as a potential therapeutic target in human cancer, with IGF-1R inhibition expected to diminish tumor cell survival [152,153]. Different cell types, including human breast cancer cells, human aortic smooth muscle cells with basal expressions of TF, human monocytes conditioned to express TF, and porcine aortic endothelial cells overexpressing human TF showed dose-dependent transactivation of the IGF-1R upon treatment with full functioning FVIIa, but not after treatment with an active site-inhibited FVIIa. IGF-1R inhibition or ablation inhibited FVIIa-mediated activation of AKT and prevented the protection of cancer cells from TRAIL-induced apoptosis [127,133,154,155,156,157,158,159]. A SUMOylated IGF-1R was also translocated to the nuclei after the treatment with FVIIa, where it worked as a transcriptional enhancer. When porcine cells were transfected with human TF containing a mutant cytoplasmic domain, TF was shown to not need phosphorylation for transactivating IGF-1R [144]. However, it is still unclear how the transmission of anti-apoptotic signals happens between TF/FVIIa and IGF-1R. It was found that there is an association between both TF and IGF-1R with domains called caveolae, which are protein-rich, persistent invaginations of the plasma membrane that operate as signaling nodes [160,161]. Recently, Åberg and colleagues demonstrated that Cav1 Tyr14 activation by Src-family kinases is caused by TF/FVIIa-dependent ITGβ1 activation. The ability of TF/FVIIa to protect cancer cells from TRAIL-induced apoptosis and stimulate IGF-1R-dependent protein synthesis was eliminated by inhibiting ITGβ1 or Src, or by Cav1 activation [162].

### 5.4. Integrin Signaling

Integrins are transmembrane cell surface receptors that play a role in various essential biological processes including cell adherence to the extracellular matrix (ECM), cell-to-cell adhesion, and cell migration. Integrins are heterodimers of non-covalently linked transmembrane α and β subunits. There are multiple distinct α- and β subunits, which are combined to form roughly 24 different integrins [163]. They play a crucial role in cell signaling by influencing multiple pathways, including the IGF-1R-induced one often resulting from their binding functions [164]. Integrins that are located on the surface of platelets are responsible for fibrin attachment within a growing thrombus during coagulation [165]. TF/FVIIa can also communicate with integrins (Figure 2B). As previously mentioned, TF/FVIIa-dependent PARs and Eph receptor signaling are activated by proteolytic cleavage. β1 integrins (ITGβ1), on the other hand, are not activated by TF/FVIIa proteolytic cleavage. However, a more direct physical connection between the complexes appears to be required for ITGβ1 activation by TF/FVIIa. The FVIIa protease domain contains an integrin-binding motif that is known to be necessary for the interaction of TF/FVIIa with ITGβ1, causing conformational changes that can lead to ITGβ1 activation. The TF/FVIIa/ITGβ1 signaling has been linked to tumor development and angiogenesis [166,167].

### 5.5. MAPK Signaling

MAPK p42/44 is documented to have a role in cell proliferation, differentiation, and survival. The MAPK p42/44 can phosphorylate a broad range of proteins, of which some immediately translocate into the nucleus. P42/44 controls several transcription factors [168]. Elk-1 is phosphorylated by p42/44, which stimulates immediate-early gene transcription. MAPK p42/44 phosphorylation promotes smooth muscle cell proliferation [169].

It was observed that upon FVIIa binding to TF, MAPK p42/44 is activated. The p42/44 inhibitor was used to inhibit phosphorylation [123,170]. However, in BHKtf cells (stable TF-transfected baby hamster kidney cells), the same mechanism is mediated through PKC (protein kinase C). Blocking Raf kinase, which is part of the traditional signaling pathway between Ras and p42/44, stopped p42/44 phosphorylation in both cell lines [170]. The PKC or Src activation by FVIIa receptor remained incompletely understood. Another study found that stimulation with FVIIa induced the phosphorylation of epidermal growth factor (EGFR) and proline-rich tyrosine kinase 2 (PYK 2) in HaCaT cells [146]. EGFR kinase inhibitors could prevent TF/FVIIa complex-mediated activation of p42/44. These inhibitors also blocked the induction of the EGR-1, hb-EGF (heparin-binding epidermal growth factor), and IL-8 genes [171]. In fibroblasts, TF was found to impact gene expression by transient activation of p44/42 MAPK and other familiar proteins such as p38 MAPK [172]. The MAPK pathway has also been linked to the overexpression of TF by VEGF, as MAPK inhibitors reduced this process [173].

## 6. The Tissue Factor Pathway and Cancer

In the past two decades of clinical research, a view of TF and its downstream pathway has emerged beyond its central role as a simple blood coagulation initiator to TF serving as a versatile signaling receptor affecting several cellular processes, including apoptosis [118], gene and protein expression [174], proliferation [175], and angiogenesis [176,177]. TF signaling events can arise both dependently or independent of its short cytoplasmic tail region. 

TF signaling recently has gained more and more attention due to its implication in several human malignancies. The first evident indication of a tight linkage between the blood coagulation cascade and pathogenesis of certain malignancies is represented by the significantly elevated thrombotic risk in advanced-stage cancer patients: 90% of metastatic cancer patients are affected by some kind of coagulopathy [178,179,180] and this characteristic prothrombotic state is responsible for a not negligible proportion of cancer-associated deaths [181]. Furthermore, TF was found to be frequently expressed and consistently upregulated in a broad range of human malignancies, pre-eminently adenocarcinomas [182]. Elevated TF expression was found inter alia in carcinomas of the bladder [183,184,185], brain [186,187,188,189,190], colon [191,192,193,194,195], ovary, and breast [196,197,198,199], and various other tissues such as lung, liver, or pancreas [200,201,202,203,204,205,206]. Additionally, research revealed a correlation between cancerous TF expression levels and the malignant potential as well as the aggressiveness of tumor cells—high TF levels were shown to be associated with a poor prognosis in a multitude of different types of cancer [185,196,207,208,209,210]. Clinical evidence underscores these findings by revealing a correlation between high TF expression levels in tumor tissues and metastasis in a variety of these types of cancer [182,194,195,211]. Together, these studies emphasize the importance of TF and its downstream targets for cancer pathogenesis. 

There are various potential causes for increased TF expression levels in cancer cells. First, oncogenic changes may impact TF expression. The upregulation of TF parallels the expression of several oncogenes in different types of cancer. For instance, TF levels were found to be elevated upon K-ras activation in human colorectal cancer cells [212]. Similarly, activation of certain members of the EGFR protein family-like EGFR and HER-2 was found to induce TF expression in multiple carcinoma cell lines [209,213,214,215] and the corresponding ligands, such as EGF and TGFα, further increased TF expression [216,217,218]. Additionally, the inactivation of the p53 tumor suppressor also led to an enhanced TF expression in human colorectal cancer cell lines [212,219]. In analogy, loss of PTEN resulted in induced TF gene expression via Akt-mTOR and Ras-MEK-ERK signaling in human glioma cell line 23.11 [219]. Clinical data from colorectal and lung cancer patients further support the correlation between mutant K-Ras and p53 and changes in TF expression [220,221]. TF levels might also be affected by the actions of particular transcription factors. Early growth response gene-1 as well as hypoxia-inducible factor-1α independently induced TF gene expression under hypoxic conditions in the human breast cancer cell lines MDA-MB-231 and MDA-MB-435 [222]. MET signaling is also involved in the link between cancer and blood coagulation. The MET gene regulates invasive growth by encoding the tyrosine kinase receptor for hepatocyte growth factor/scatter factor [223]. Under hypoxic conditions, which are common in the central areas of solid tumors, this oncogene is activated [224]. The transcription of the hemostasis genes including plasminogen activator inhibitor-1 (PAI-1) and cyclooxygenase-2 (COX-2) is induced by activated MET oncogene, which results in the polymerization of fibrin around tumor cells, providing a scaffold for angiogenesis [223]. Thrombo-hemorrhagic syndrome can also be caused by an activated MET oncogene, as well as activated PAI-1 and COX-2 [223]. Epithelial–mesenchymal transition (EMT) is a critical step during the establishment of metastases in advanced disease stages. Centrally involved transcription factors Snail and ZEB1 also were shown to induce TF expression in breast cancer cells [225]. Additionally, both AP-1 and NFκB transcription factors were shown to induce TF expression in the human breast cancer cell line MDA-MB-231 [226]. 

Notably, the fact that both mutant K-Ras as well as p53 inactivation, the two most common genetic alterations in human malignancy [212], can induce and elevate TF expression, respectively, emphasizes the frequency of this condition and therefore underlines its significance for cancer pathogenesis. 

There is growing evidence suggesting TF as a key regulator rather than an incidental participant regarding several cellular events in cancer progression. Since the dawn of the new millennium, numerous studies have attributed the role of TF to cancer progression and tumor growth. As discussed in detail above, the activation and action of the coagulation cascade comprise various proteases. Besides their canonical role for proper blood clotting following vessel injury, these proteases are also able to cleave the extracellular domains of certain PAR proteins, ultimately triggering G protein- and β-arrestin-coupled signaling, respectively [227,228,229]. Additionally, PAR1 might be partially activated also by FXa as well as aPC, MMP-1, and MMP-13 [230,231,232].

**Figure 3:**
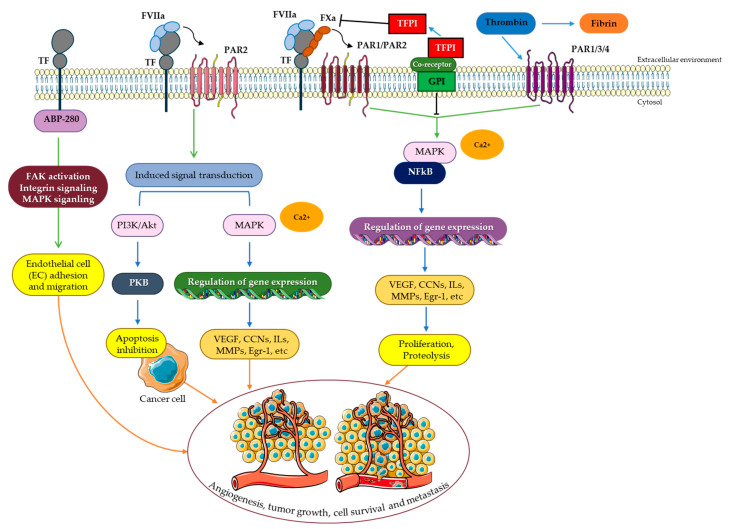
Possible mechanisms of expression and functional regulation of tissue factor and coagulation factor VII in cancer cells. Schematic overview summarizing the multiple mechanisms of gene expression, functional tuning, and intracellular signaling in cancer cells, as described in this review. It has been suggested that cancer cell phenotypes associated with the TF-fVII pathway may be specifically controlled via multiple autocrine and/or paracrine mechanisms, depending on tumor microenvironmental conditions. Parts of the figure were drawn using pictures from https://smart.servier.com/ (accessed on 16 May 2022).

In cancer cells, PAR2 activation by TF/FVIIa was found to induce Akt phosphorylation and inactivation of glycogen synthase kinase-3b (GSK-3b), finally resulting in the upregulation of the Wnt pathway (Figure 3) [126,233,234]. These results are further encouraged by studies on breast carcinoma cells [196,235]. In another study, the promigratory effect as a consequence of TF/FVIIa-dependent PAR2 signaling was found to be more indirect due to the release of interleukin-8 (IL-8) [236]. The cytokine binds G protein-coupled receptors (GPCRs) CXCR1 and CXCR2 [237,238] and affects several cellular processes. Effects on cell migration are postulated to be partially mediated by CXCR-dependent activation of particular members of the Rho-family of GTPases, ultimately resulting in actin cytoskeleton rearrangements [239,240].

Beside GPCR signaling, PAR cleavage might also induce a G protein-independent pathway via the recruitment of β-arrestin. This intracellular molecule takes a prominent role in membrane receptor internalization [241] and is postulated to promote breast cancer migration through the cofilin pathway [242,243]. Blocking either PAR2 or specifically, the signaling functions of the TF/FVIIa binary complex suppressed tumor growth in a xenograft model in immunodeficient mice, while inhibition of the TF-initiated coagulation only exhibited a minute effect [167,244], accentuating the importance of TF-PAR2 signaling for cancer progression [245,246]. 

### 6.1. The Tissue Factor Pathway and Cancer-Induced Hypercoagulability

In cancer patients, hypercoagulability increases the risk of venous thromboembolism (VTE), pulmonary embolism (PE), disseminated intravascular coagulopathy (DIC), and bleeding [247]. Furthermore, hypercoagulable conditions can foster carcinogenesis by inducing angiogenesis. As documented in human breast cancer cell lines, TF is required for hypercoagulability as the clot formation is FVIIa-dependent and could be inhibited by anti-TF antibodies [248]. Upregulation of TF is also linked to VTE in pancreatic cancer and serves as an independent predictor of VTE in ovarian cancer [198,204,249]. Additionally, overexpression of TF has been shown to result in the systemic production of TF-positive extracellular vesicles, which can be used as a predictive marker for deep vein thrombosis (DVT) [250]. It was also reported that TF-positive extracellular vesicles can be correlated with D-dimer levels which significantly predict VTE in cancer patients [191].

Patients with cancer are more likely to experience thromboembolic events, particularly those receiving chemotherapy and radiotherapy. Every year, 11% of cancer patients undergoing chemotherapy develop VTE, and hormone treatments (such as tamoxifen) raise the risk by two- to three-fold [251]. Furthermore, anti-angiogenic medications are unexpectedly linked to a high rate of thrombosis, although these findings could be biased because several clinical trials combined antiangiogenics and chemotherapeutics [252]. Despite the cancer treatments appearing to influence the hypercoagulability of the patients, the mechanisms are still uncertain. It was previously demonstrated that an elevation in the tumor-secreted extracellular vesicles and cell-free DNA can contribute to this condition [253]. In vitro treatment of endothelial cells with a VEGFR-2 inhibitor in combination with chemotherapy (especially cisplatin and gemcitabine) resulted in a significant elevation in surface TFPI expression, indicating extracellular procoagulant transformation; however, this transformation was diminished when the chemotherapeutic treatments were prescribed at a lower dose [254,255].

### 6.2. The Tissue Factor Pathway and Angiogenesis

TF-related angiogenesis arises both indirectly and directly through the coagulation or release of proangiogenic factors [256,257,258]. TF-induced thrombin production is partially responsible for the coagulation-associated indirect control of angiogenesis. The fibrin clot that is formed provides a proangiogenic matrix that promotes blood vessel invasion [257]. Moreover, coagulation causes proangiogenic factors to be released from the granules of active platelets. This activates other angiogenic pathways that are reliant on FXa production, thrombin, and the signaling of PARs [259]. Additionally, there has recently been emerging experimental and early clinical evidence indicating TF-dependent PAR signaling to forward tumor progression by promotion of proangiogenic processes either through upregulation of proangiogenic factors such as VEGF or downregulation of anti-angiogenic molecules like thrombospondin (Figure 3) [212,219,245,256,258,260]. The evocation of new blood vessels enabling cancer cells to access the circulatory system and simultaneous regression or even collapse of the pre-existing vasculature in close vicinity are critical steps during tumor growth due to the reliance of cancer cells on metabolic exchange and oxygen as well as access to vascular and lymphatic channels for efficient intravasation [261,262,263]. In breast cancer cells, TF/FVIIa-mediated PAR2 cleavage and subsequent signaling events were shown to induce proangiogenic factors such as VEGF [264], Cyr61, CXCL1, and IL-8 [136,236]. Biopsies of newly diagnosed invasive breast cancer patients revealed a significant increase in TF and PAR2 compared to non-invasive ductal carcinoma in situ paralleled an elevated VEGF expression, emphasizing the link between tumor angiogenesis and TF-dependent PAR2 signaling in a more clinical model and further in vivo results manifest this impression [228,265]. Besides the initial TF/FVIIa complex, the thrombin molecule also settled more downstream in the blood clotting cascade, positively affecting tumor angiogenesis [266,267,268]. Local thrombin generation as a consequence of the action of the TF pathway may upregulate VEGF receptor expression through paracrine PAR1 activation in stromal cells or tumor cell PAR1 signaling in an autocrine manner [269], potentially enhancing responsiveness of these cells to VEGF stimulation. Further evidence for TF-mediated effects on cancerous angiogenesis was provided by animal studies in which specific inhibition of TF/FVIIa suppressed tumor growth and in vivo angiogenesis [256]. Other studies indicate that the expression of anti-angiogenic thrombospondins is substantially repressed in TF-positive tumor cells [212,258], which might additionally enhance the actions of proangiogenic growth factors. Since fibrinogen serves as a scaffolding molecule for cell migration as well as the binding site for promigratory and angiogenic factors such as VEGF, ECM remodeling events following thrombin activity might also impact angiogenesis in the tumor tissue and its microenvironment [180]. TF/FVIIa promotes p44/42 MAPK as well as Akt/protein kinase B phosphorylation in breast cancer cells, which in turn triggers the mTOR pathway that is involved in the regulation of several cancer-related processes such as cell survival, growth, proliferation, and motility [270].

### 6.3. The Tissue Factor Pathway and Cancer Cell Survival

Apoptosis resistance is a well-known strategy for malignant cell survival, as well as the induction of anti-apoptotic pathways, and the occurrence of defects in apoptosis promote carcinogenesis and metastasis [271]. As mentioned previously, FVIIa is a well-known activator of the anti-apoptotic signaling pathways involving P42/44 MAPK and protein kinase B [58]. Interestingly, previous studies, performed on TF-overexpressing human breast cancer cells (MCF-7), showed that the formation of the TF-FVIIa complex inhibited apoptosis in a thrombin-dependent manner by affecting the phosphorylation of both P42/44 MAPK and protein kinase B/Akt and causing stimulation in anti-apoptotic survivin expression (Figure 3) [270].

Additional findings demonstrated that supplying FVIIa to serum-depleted baby hamster kidney and Chinese hamster ovary cells expressing TF improved cell viability [162]. Furthermore, since TF-FVIIa signaling has been documented to be involved in the creation of STAT5-dependent BclxL and Jak2-dependent protein kinase B activation, lack of adhesive ability may be the cause of the anti-apoptotic characteristics mediated by FVIIa [272]. Protection against immune response and cytotoxicity is another strategy by which TF improves tumor cell survival. The TF cytoplasmic domain was found to be responsible for a 40% invasion frequency from peripheral blood monocytes in TF-expressing colon cancer cells [273,274]. Although the exact mechanism is still unknown, this could contribute to enhanced cell survival and metastasis.

### 6.4. The Tissue Factor Pathway and Metastasis

The coagulation cascade, particularly thrombin release, is critical for metastasis. TF has been shown to have an important function in attenuating the attacks of natural killer cells to forming or still established micrometastases by fibrinogen-dependent and platelet-dependent mechanisms [275]. Moreover, fibrinogen displays an important role in metastasis due to its central involvement in tumor cell dissemination to establish metastases via the lymphatic or hematogenous system. Remarkably, non-metastatic breast cancer cells have limited TF expression, whereas metastatic breast cancer cells display abundant cell surface TF. However, the mechanisms remain unclear [273]. 

Perhaps the downstream coagulation factors affect the production of TF-FVIIa and play a role; nonetheless, a promising idea implies that TF-FVIIa signaling enhances cell motility. TF-FVIIa signaling has been linked to cell motility in numerous studies. In vitro, cleaving of the extracellular domain of TF caused the TF cytoplasmic domain to interact with actin-binding protein 280 (ABP-280), which is implicated in cell migration and adhesion [276]. Furthermore, the PARs have been linked to tyrosine kinase receptors in a previous study, correlating TF-FVIIa signaling to the migration caused by the platelet-derived growth factor-BB [126]. In invasive breast tumors, PAR1 is extensively expressed and activated by thrombin to increase their invasiveness, but it is not found in healthy breast tissue or non-invasive malignancies. In addition, thrombin-dependent PAR1 signaling was also found to influence tumor cell motility. The cytoplasmic domain of TF is required for migration in human bladder carcinoma [172]. Experimental tumor cell metastasis, besides TF/FVIIa-driven thrombin generation, was dependent on the signaling properties of the TF intracellular domain (Figure 3) [277,278]. However, further studies with contradictory results have been published. PAR1 signaling in both tumor and host cells was credited essentiality for TF-dependent lung metastasis of breast cancer cells and the PAR signaling pathway was postulated to enhance breast cancer cell invasiveness and tumorigenesis [279]. PAR2 partially contributes to thrombin-PAR1 signaling-dependent tumor cell metastasis via cross-activation [280]. On the other hand, by cleavage of the tyrosine kinase receptors EphA2 and EphB2, TF/FVIIa complex can increase cell migration [143]. MiR-19 decreases TF expression in breast cancer cells similarly, and the data suggest that miRNA control of TF may alter tumor-associated processes [281]. In colon cancer cells, miR-19a decreased TF production and thus migration and invasion, demonstrating a more serious influence of miRNA on TF activity [282]. The metastatic potential of melanoma cells implanted into severe combined immunodeficiency mice was found to be correlated with the level of TF [277]. Furthermore, suppressing TF with anti-TF antibodies, siRNA, and the Fab fragment of TF antibodies all were documented to decrease melanomas and breast cancer metastasis [283,284,285]. Notably, both cytoplasmic TF signaling and the cell surface proteolytic activity of TF-FVIIa were considered to be significant in this context [277,284].

### 6.5. Impact of TF Domains and Complexity of TF and Its Effector FVIIa on Cancer Progression

Apart from TF-dependent effects on cancer progression via cleavage of particular PAR proteins, the transmembrane receptor might also influence certain tumor-associated processes more directly. First, the large extracellular domain of the TF is known to impact pathways relevant to cell adhesion processes, primarily through interaction with several integrins through which it might influence migratory and invasive properties as well as signaling pathways of cancer cells [286,287]. 

Surprisingly, despite its brevity of just 21 residues, the cytoplasmic tail region is said to affect certain processes with an essential role in cancer progression and tumor growth. TFt provides two potential phosphorylation sites at Ser253 and Ser258, which can be phosphorylated by PKC [132,286] and MAPK p38 [288], upon extracellular binding of FVIIa and resulting conformational changes [289,290]. Phosphorylated TF cytoplasmic tail is postulated to serve as a binding site for actin crosslinking protein filamin A whose recruitment is dependent on the complexation of TF and FVIIa [276,291]. Filamin A is predicted to have a tumor-promoting role when localized to the plasma membrane or cytoplasm due to its diverse role in cell migratory and adhesive processes following the interaction with various signaling molecules [292,293]. This relationship is further manifested by another study finding that the short cytoplasmic region may act as a positive regulator of tumor growth under certain conditions [245]. 

Numerous studies implicate FVIIa-dependent TF cytoplasmic tail signaling in tumor metastasis [277], angiogenesis, and corresponding vascular remodeling events [172,265], as well as inflammatory and immune responses [12,294]. Deregulated TF phosphorylation contributes to the aggressive behavior of invasive tumor cells [295]. Cytoplasmic tail signaling may also interplay or even synergize with coagulation cascade component-mediated PAR signaling [17]. Phosphorylation of the TF cytoplasmic region was evidenced to contribute to TF/FVIIa-dependent PAR2 signaling and tumor growth in a murine model system [135]. In this regard, clinical data from patients with recurrent breast cancer indicated an association between TF cytoplasmic tail phosphorylation and PAR2 expression [296]. 

Data from numerous studies indicate the assembled TF/FVIIa tandem contributes to tumor growth and tumor-favoring alterations in the TME in a variety of different types of cancer in experimental but also preclinical models [167,256,297] orchestrated by a plethora of cellular mechanisms [212].

Notwithstanding all these studies, the question remains how TF and its effector FVIIa may even be complex in tumor tissues in the first place since TF is still located extravascularly while its downstream proteases circulate in the blood. First, coagulation factors such as FVIIa or FXa might be able to readily enter the tumor from the blood due to the leaky tumor vasculature, which allows complex assembly on the tumor cell surface [298]. Moreover, following migration and intravasation, cancer cells might also gain access to the bloodstream allowing direct interaction of surface TF with circulating effector proteases. Additionally, oncogenic pathways might also stimulate the release of TF-containing microvesicles (MVs) from cancer cells into the circulatory system [215,299]. Last but not least, circulating TF can also be found in its soluble form as a cleavage product of full-length TF or asTF [23,300,301,302,303], in both cases lacking the transmembrane domain and the cytoplasmic tail. Circulatory asTF, despite the absence of the regulatory cytoplasmic region as well as displaying no membrane association, has also been shown to enhance tumor growth and cancer angiogenesis [302,304,305,306], although the responsible mechanism has not yet been fully elucidated. 

## 7. Tissue Factor Pathway Inhibitor and Cancer

In accordance with these potentially tumor-promoting properties of TF, its natural inhibitors TFPI-1 and TFPI-2 have been described to act as tumor suppressors [307]. It was reported that the downregulation of TFPI decreased the apoptosis in breast cancer cells, but ectopic overexpression of TFPI increased apoptosis [308]. Moreover, stable downregulation of both isoforms of TFPI was found to increase the metastatic growth of breast cancer cells by boosting cell proliferation, motility, and invasion [309]. Enhanced MMP-2 and MMP-9 activity were observed, which could clarify the increase in cell invasion. Elevated integrin levels may indeed promote the tumor cells to be more aggressive. Downregulation of TFPI improved integrin-mediated adhesion and cell proliferation in MDA-MB-231 cells, as evidenced by increased cell adhesion and stress fiber formation in response to an ECM constituent [309]. Furthermore, it was assumed that TFPI knockdown increased the expression and activity of collagen-binding integrins, which was contrary to Sum102 in terms of which there was no difference in adherence to collagen I [310]. Notably, the downregulation of TFPIβ but not TFPIα was observed to enhance the cell motility in MDA-MB-231 cells [309]. Overexpression of TFPI was recently known to trigger apoptosis in SK-BR-3 breast cancer cells [308].

TFPI-2 is frequently downregulated in the vast majority of aggressive tumors such as glioma [311], breast cancer [312], melanoma [313], colorectal cancer [314], and pancreatic cancer [315], just to name a few. Downregulation mainly relies on epigenetic alterations in cancer cells, primarily hypermethylation of TFPI-2 promoter or histone deacetylation [312,316,317,318]. Low or absent TFPI-2 expression in breast cancer patients was associated with increased metastatic growth and angiogenesis and thus with advanced disease progression, recurrence, and poor survival outcome [307,309]. Similar correlations were found in a multitude of other different types of cancer such as gastric cancer [319], melanoma [320], and glioma [321], as well as nasopharyngeal [322] and oesophageal carcinoma [317]. Following these findings, it was suggested that restoration of TFPI might potentially be of antitumoral value due to the diminishment of cancer aggressiveness. Kondraganti and colleagues found that the reintroduction of TFPI-2 inhibits tumor invasion and growth in vitro and in vivo in a malignant melanoma cell line [323]. Similar results were published for prostate cancer cells [324] and glioblastoma cells [325], as well as oesophageal and pancreatic carcinoma cells [326]. TFPI-2 antitumoral effects are further strengthened by its capability to decelerate cell proliferation by inducing apoptosis in small-cell lung cancer (SCLC) [327] and a glioblastoma cell line [325]. Peritumoral TFPI injection further was demonstrated to suppress tumor growth in melanoma, even though this effect was only temporary [256]. Additionally, intravenous injection of recombinant TFPI in mice directly after injection of B16 mouse melanoma cells significantly reduced experimental lung metastasis [320]. These gathered data together indicate a potential therapeutic value of TFPI regarding substantial inhibition of invasiveness and aggressiveness of certain different types of cancer and thus deceleration of cancer progression. 

Nevertheless, the role of TFPI in cancer progression and tumor growth is not as unambiguous as the first impression suggests it to be. Its understanding becomes more challenging when taking into account that TFPI was shown to bind ECM components, thus supporting the TF/FVIIa complex to promote tumor cell adhesion and migration [328]. Therefore, it remains to be tested whether the clinical applicability of TFPI is a potential antimetastatic strategy [329].

## 8. TF, TFPI, and Proteoglycans

Proteoglycans (PG) are glycoproteins characterized by the covalent attachment of one or more carbohydrate chains of the glycosaminoglycan (GAG) type [330]. Among the different classes of these highly negatively charged non-branched linear chains of repetitive disaccharide units, heparan sulfate (HS; N-acetylglucosamine-α-L-iduronic acid/β-D-glucuronic acid) is of particular relevance for this review due to its structural similarity to the anticoagulant heparin. PGs, collagens, and additional non-proteoglycans are known to make up the vascular ECM, which is thought to isolate the layer of endothelial cells from circulating blood [331]. However, the mechanism by which heparin enters ECM and quickly redistributes TFPI from ECM into circulating blood is still unknown. TFPI released by heparin can be found in vascular beds with fenestrated endothelium, such as the liver and bone marrow [332]. Heparin perfusion, on the other hand, released TFPI confined to the ECM surrounding the umbilical vein, demonstrating that the release also happens in circulatory beds without fenestrated endothelium [332].

It was also reported that in vivo infusion of heparin induced a two- to four-fold increase in the level of circulating TFPI [333]. The lack of increase in TFPI concentration ex vivo, and the addition of heparin to plasma, suggests the release of TFPI from intracellular or extracellular stores, such as to HS or other GAGs of the endothelium, for instance, dermatan sulfate or chondroitin sulfate C to be released [334]. This bound form of TFPI of complete length appears to possess, in principle, a higher inhibitory capacity to FXa, which can be additionally enhanced by heparin than the “COOH-cleaved” TFPI forms circulating in plasma [332]. 

GAGs appear to be the favorite molecules for TFPI accumulation at the endothelial cell surface. The following facts support this hypothesis suggesting that TFPI interacts with heparin agarose [335]; heparin and sulfated polysaccharides improve the anticoagulation capability of TFPI [336]. TFPI plasma levels rise several-fold following intravenous heparin administrations, and heparin contends for numerous binding sites of TFPI on the cell surface [84,337].

TFPI-1 is secreted on stimulation by thrombin and is thus able to participate in the control of thrombus formation by its increased concentration at the site of vascular injury [338]. Inflammatory mediators induce TF in vessel wall cells and monocytes but have no significant effect on the synthesis of TFPI-1 [339,340]. 

Proteoglycan receptors shown to bind TFPI-1 include the transmembrane ryudecan/syndecan 4 (SDC-4) and the glycosylphosphatidylinositol (GPI)-anchored glypican 3 (GPC-3), with the type of membrane anchoring critically influencing the respective localization of surface receptors on specific regions of the cell membrane [341]. GPC-3 in liver cells and SDC-4 isolated from endothelial cells have been found to bind to TFPI [87,88]. TFPI-1 is associated with HSPGs because of the Kunitz domain 3 and the C-terminal end, which are essential for effective binding to cell surfaces of endothelium and hepatoma cells [342,343]. Moreover, the evidence stating that endogenous TFPI-1 is mainly anchored on the surface of ECV304 cells via a GPI linkage and investigations employing the primary HUVECs in culture came up with similar results. 

Furthermore, a large pool of TFPI-1 is non-covalently linked to endothelial cell HSPGs such as syndecan-1 (SDC-1) in quantities two to four times the plasma concentration [344]. SDC-1 levels in patients suffering from thermal injuries were found to be potentially considered as a marker of endothelium damage associated with age and increased 24 h fluid needs in trauma patients, but not with burn size or death, according to a previous study [345,346]. 

Furthermore, it was shown that burn injury causes SDC-1 shedding, and that plasma-based restoration can reduce vascular leakage in a rat model of burn injury [347]. Plasma TFPI levels may be raised as a result of endothelial binding site loss associated with endothelium SDC-1 shedding, platelet release, or both [348]. Furthermore, heparinase or heparitinase, but not chondroitinase treatment, significantly inhibited FXa-stimulated-TFPI uptake and degradation. Growing the cells in chlorate-containing media inhibited GAG sulfation, which reduced FXa-stimulated-TFPI breakdown. These findings imply that HSPGs are necessary for TFPI/FXa complex uptake and breakdown [349].

On the other hand, Nassar et al. observed that silencing of SDC-1 reduced HUVEC tubule network formation in triple-negative breast cancer (TNBC) cells. Moreover, in the SDC-1-silenced secretome, the angiogenesis array indicated lower levels of VEGF-A and TF. SDC-1 depletion resulted in lower secreted endothelin-1 (EDN-1) and TF levels, while stimulation with TFPI inhibited angiogenesis. These findings imply that SDC-1 may control the angiogenesis of cancer cells through the TF pathway in addition to other angiogenic pathways [350].

## 9. Functional Enrichment Study of Tissue Factor Pathway Targets with HSPG-Related Proteins on the Cell Surface

As mentioned in the previous chapter, experimental evidence for TF and TFPI interactions with GAGs and HSPGs exist, but a generalization or a valid basis for this finding is still not fully understood. TF interacts with a range of signaling receptors, which trigger the activity of several signaling pathways and biological processes ranging from coagulation to malignancy including cell survival, inflammation, and angiogenesis. HSPGs as part of the ECM play a role in both physiological and biochemical processes, such as signaling during inflammation and the stimulation of cell proliferation, adhesion, motility, angiogenesis, and tumor growth [351]. The capability of HSPGs to control the expression and activity of growth factors, cytokines, chemokines, and adhesion molecules is one of their most significant features. This is owing to their ability to function as co-receptors for a variety of signaling molecules, including FGF, VEGF, integrins, Wnts, IL-6/JAK-STAT3, NFkB, and others [351]. In light of the diversity of findings and the diversity of approaches, we aimed to generate a general virtual approach using STRING analysis. We explored the protein interactions in silico using the free online STRING database to display the interaction network between HSPGs and factors involved in the TF pathway [352]. The STRING database serves as an online tool that exports identified pathways, interaction predictions, and protein networks from curated resources to obtain protein–protein interactions [352]. 

**Figure 4:**
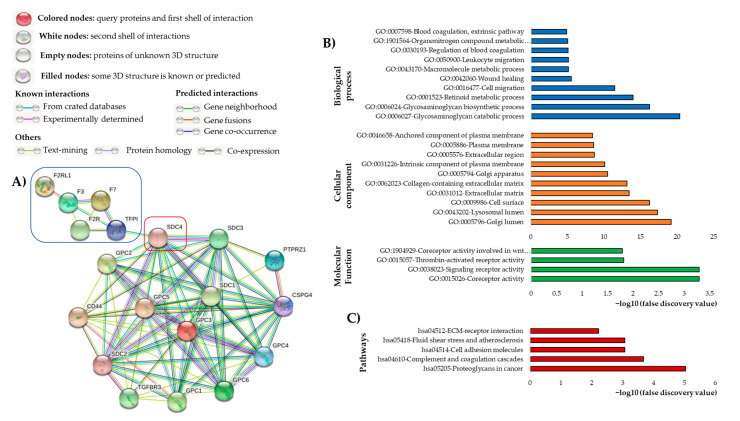
Network of proteoglycans with tissue factor pathway interactors. (**A**) STRING database output depicting functional and physical interactors of TF pathway-related proteins (in the blue box) and HSPGs obtained from http://string-db.org/ (accessed on 16 May 2022) [352]. Interactions with a combined score > 0.700 were defined as statistically significant. (**B**) GO (gene ontology) analysis of the interconnection between HSPGs and TF pathway-related pathways. The 10 most significantly (*p* < 0.05) enriched GO terms in molecular function (green), cellular component (orange), and biological process (blue) branches are presented. (**C**) KEGG pathway analysis (red). All the adjusted statistically significant values of the terms were negative 10-base log-transformed. The color coding of lines and nodes is shown in the figure.

Figure 4A demonstrates the interaction of targets of tissue factor pathway (highlighted blue box) with cell surface HSPGs, showing that (a) TF and other targets involved in thrombosis and coagulation process along with TFPI are highly and closely interconnected whereas TFPI directly and strongly interacts with SDC-4 cell surface HSPG; and (b) almost all HSPGs including syndecans (SDC-1, 2, 3, 4), glypicans (GPC-1, 2, 3, 4, 5, 6), and CD44, betaglycan (TGFBR3), CSPG4 and phosphacan (PTPRZ1) are strongly and directly interconnected.

As shown in Figure 4B, this complicated protein network is engaged in various key molecular and biochemical functions, cellular components, and biological processes (green, orange, and blue bars, respectively). Particularly, the receptor and co-receptor activity, thrombin-activated receptor activity, and coreceptor activity were observed to be involved in Wnt signaling planar cell polarity pathways within the molecular function category. Regarding the cellular component category, the HSPGs and TF pathway-related proteins are associated with the Golgi and lysosomal lumen, cell surface, extracellular matrix, collagen-containing extracellular matrix, an intrinsic component of the plasma membrane, extracellular region, and anchored component of the plasma membrane. On the other hand, the biological process category to which TF pathway-related proteins and HSPGs are related include glycosaminoglycan catabolic and biosynthetic processes, retinoid metabolic processes, cell and leukocyte migration, wound healing, macromolecule metabolic processes, blood coagulation regulation, organonitrogen compound metabolic processes, and blood coagulation (extrinsic pathway).

KEGG pathway findings show biological pathways related to proteoglycans in cancer, and atherosclerosis, as well as complement and coagulation cascades, cell adhesion, ECM-receptor interaction, and fluid shear stress, confirming the analysis of the gene ontology (GO) (Figure 4C).

It appears that the network of interactions between HSPGs and TF pathway-related proteins, as well as the potential effects of those interconnections, are important not only for maintaining normal cell function but also for inducing carcinogenesis and malignancy. As a result, understanding more about the relationships of these HSPGs with TF pathway-related proteins may enhance our insight into the mechanisms through which these molecules might be useful targets for cancer therapy. We can also demonstrate from these analyses that TFPI emphasizes as the maestro of this interconnection, due to its direct interaction with SDC-4, linking the HSPGs and the TF pathway together. In previous experimental work, it was already shown that HSPGs on endothelial cells might function as receptors for the internalization of TFPI-FXa complexes, contributing to the anticoagulant TFPI activity. On another hand, Tinholt et al. observed the association between SDC-3 and endogenously expressed TFPI. FGF, heparin-binding growth-associated molecule, EGFR, and notch signaling ligands are considered to act as SDC-3 co-receptors for various growth factors and ECM components [353].

Since a reduction of cell surface-related-TFPI antigen was found in GPC-3 knockdown cells, the GPI-anchored molecule might be engaged with TFPI binding [354]. This result is supported by the previous finding that TFPI binds to GPC-3 in the HepG2 tumor liver cell line [355]. The connection between TFPI and GPC-3 could be a mechanism for eliminating the TFPI-FXa complex from circulation.

## 10. Conclusions

In the last decades, the role of TF has been expanded beyond its central role as a simple blood coagulation initiator. Notably, the dysregulation of TF pathway constituents in several tumor entities and TF-associated signaling functions suggest an important mechanistic role for this pathway in tumor progression. Cancer-induced hypercoagulability, apoptosis (resistance), tumor angiogenesis, and metastatic spread are important clinicopathological processes affected by the TF pathway. While complex context-dependent effects exist, TF exhibits many tumor-promoting properties, whereas its natural inhibitors TFPI-1 and TFPI-2 have been associated with tumor suppressor activity. Notably, HSPGs add to the mechanistic complexity, as they are necessary for TFPI/FXa complex uptake and breakdown. Moreover, recent results suggest a role for some HSPGs in regulating the expression of TF constituents, resulting in altered tumor angiogenesis. These findings stimulate new ideas and may expand the possibility of targeting of selected TF pathway functions in malignant diseases in the near future.
